# Intermittent Diplopia and Dyspnea: The Subtle Face of a Cardiac Myxoma

**DOI:** 10.7759/cureus.82537

**Published:** 2025-04-18

**Authors:** Zachary R Spahr, Kyle Bruun, Mostafa Vasigh, Debanik Chaudhuri

**Affiliations:** 1 Internal Medicine, MedStar Georgetown University Hospital, Washington DC, USA; 2 Internal Medicine, Walter Reed National Military Medical Center, Bethesda, USA; 3 Interventional Cardiology, University of Minnesota, Minneapolis, USA; 4 Interventional Cardiology, State University of New York Upstate Medical University, Syracuse, USA

**Keywords:** atrial fibrillation, diplopia, exertional dyspnea, myxoma, syncope

## Abstract

Cardiac myxomas are rare primary cardiac tumors that present with clinical features that mimic a broad range of pathology; therefore, understanding the manifestations of the disease is essential for prompt diagnosis. In this case, a 53-year-old male presented after an episode of presyncope, with a six-month history of diplopia, diffuse finger and toe pain, exertional shortness of breath, cyclical fevers, and fatigue. It is a subtle, nonspecific presentation more suggestive of rheumatological or neurological disease than a primary cardiac pathology. MRI of the brain showed no acute pathology, but further workup revealed elevated inflammatory markers, pulmonary congestion, and rSr' pattern on EKG, suggesting possible right ventricular (RV) strain or conduction system disease. To evaluate for structural heart disease causing presyncope, an echocardiogram revealed a left atrial mass that was excised with resolution of symptoms.

## Introduction

Primary cardiac tumor is a rare phenomenon that occurs in approximately 0.02% of the population [1]. Myxoma comprises 50% of primary cardiac tumors, with 90% found in the left atrium [2]. When promptly diagnosed, surgical outcomes are excellent (0.7-3.5% early in-hospital mortality) with low recurrence rates (1.5-5%) [3,4].

The key diagnostic test for myxomas is echocardiography; however, the test can be delayed due to myxoma's nonspecific clinical presentation mimicking neurological, rheumatological, or infectious pathology. For this reason, clinical diagnosis is rare, at only 5.7% in one study, owing to the nonspecific symptomatology and degree of overlap with autoimmune/infectious/rheumatological pathology [5].

Myxomas have varied clinical presentations. Most studies agree cardiopulmonary (dyspnea, angina, palpitations) symptoms are most common due to disruption of flow through the mitral valve, followed by neurologic changes (focal weakness, diplopia), secondary to embolization of the primary tumor fragments, and constitutional symptoms, including fever, weight loss, arthralgias, and Raynaud’s phenomenon, thought to be secondary to the interleukin-6 (IL-6) elevation [3-8].

IL-6 elevation also leads to C-reactive protein (CRP) and erythrocyte sedimentation rate (ESR) elevations, often seen in these patients [6-9]. Ischemic cerebral infarct is the most common neurologic manifestation in myxoma, and among patients with neurologic abnormalities, imaging is almost always able to identify infarction. The ischemic infarctions follow a multi-territorial cardioembolic pattern [10-12].

On chest X-ray, clinicians may appreciate cardiomegaly, pulmonary edema, or a cardiac silhouette indicative of left atrial dilation, all of which are secondary to obstructive symptoms of the tumor. An echocardiogram typically reveals a mobile mass arising from the atrial septum, although less commonly, it may arise from the posterior wall, anterior wall, or appendages [2]. Transesophageal echocardiogram (TEE) is slightly more sensitive (96.8%) than transthoracic echocardiogram (TTE) (93.3%) in the diagnosis of atrial myxoma [13]. Chest CT has been shown to have a slightly worse sensitivity of 88.8% [14].

## Case presentation

A 53-year-old Caucasian male with a past medical history of hypertension and paroxysmal atrial fibrillation, not on anticoagulation due to CHADS2-VASc score of 1, initially presented to the ED due to persistent episodes of binocular diplopia associated with nausea and vertigo. These episodes started three months prior, typically lasted five minutes, and were self-resolving.

Further history revealed that the patient had been experiencing fever and chills for the past week. Over the previous months, he reported varying symptoms that included migratory visual field defects, reddening of his neck and cheeks with sun exposure, left arm numbness and pain, pain in his fingers and toes bilaterally with associated redness, and shortness of breath. The exam at that time was negative for significant rash or joint swelling.

Workup of the patient’s nonspecific symptoms of shortness of breath and episodes of dizziness had included pulmonary function tests (PFTs), which failed to unveil an etiology for the patient’s ongoing complaints; diffusion capacity was not included, and no obstructive or restrictive pattern was appreciated. Of note, he had undergone a stress echocardiogram in 2017, which had shown above-average exercise capacity with a Duke stress test score of 15, and a negative stress test by ECG and echocardiographic criteria with only mild mitral regurgitation (MR) without any other reported structural abnormalities. Despite negative Dix-Hallpike and lack of positional provocation of his episodes, he was diagnosed with benign paroxysmal positional vertigo due to the temporal nature of his vertigo.

**Figure 1:**
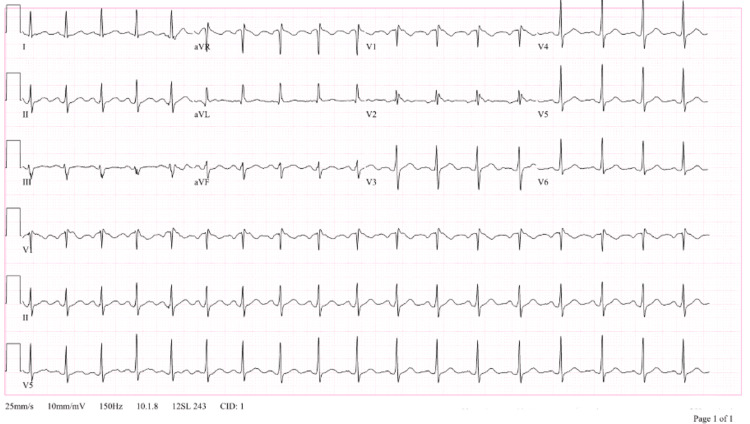
EKG findings showing sinus rhythm with normal atrioventricular (AV) conduction and rSr’ pattern.

Initial ECG showed sinus rhythm with an rSR’ pattern (Figure 1), and physical exam revealed no acute neurologic abnormalities. The ECG finding was initially thought to be a benign variant, although right ventricular (RV) strain or conduction disease could not be excluded. Neurology was consulted, and the patient was found to have an elevated CRP and ESR (Table 1) along with an unremarkable brain MRI and brain magnetic resonance angiography (MRA). Given his symptoms with no specific neurological localization, a broad range of differential diagnoses were considered, including systemic lupus erythematosus (SLE), transient ischemic attack (TIA), functional disorder, tick-borne illness, and myasthenia gravis. TIA was considered to be less likely as the symptoms were viewed as occurring in an atypical stroke distribution. The patient was discharged and advised to follow up with an outpatient rheumatology workup.

**Table 1:** Biochemical and metabolic panel. CRP: C-reactive protein; ESR: erythrocyte sedimentation rate.

Assay	Result	Units	Reference range
CRP	105.7	mg/L	0-10
ESR	29	mm/hr	0-15

**Figure 2:**
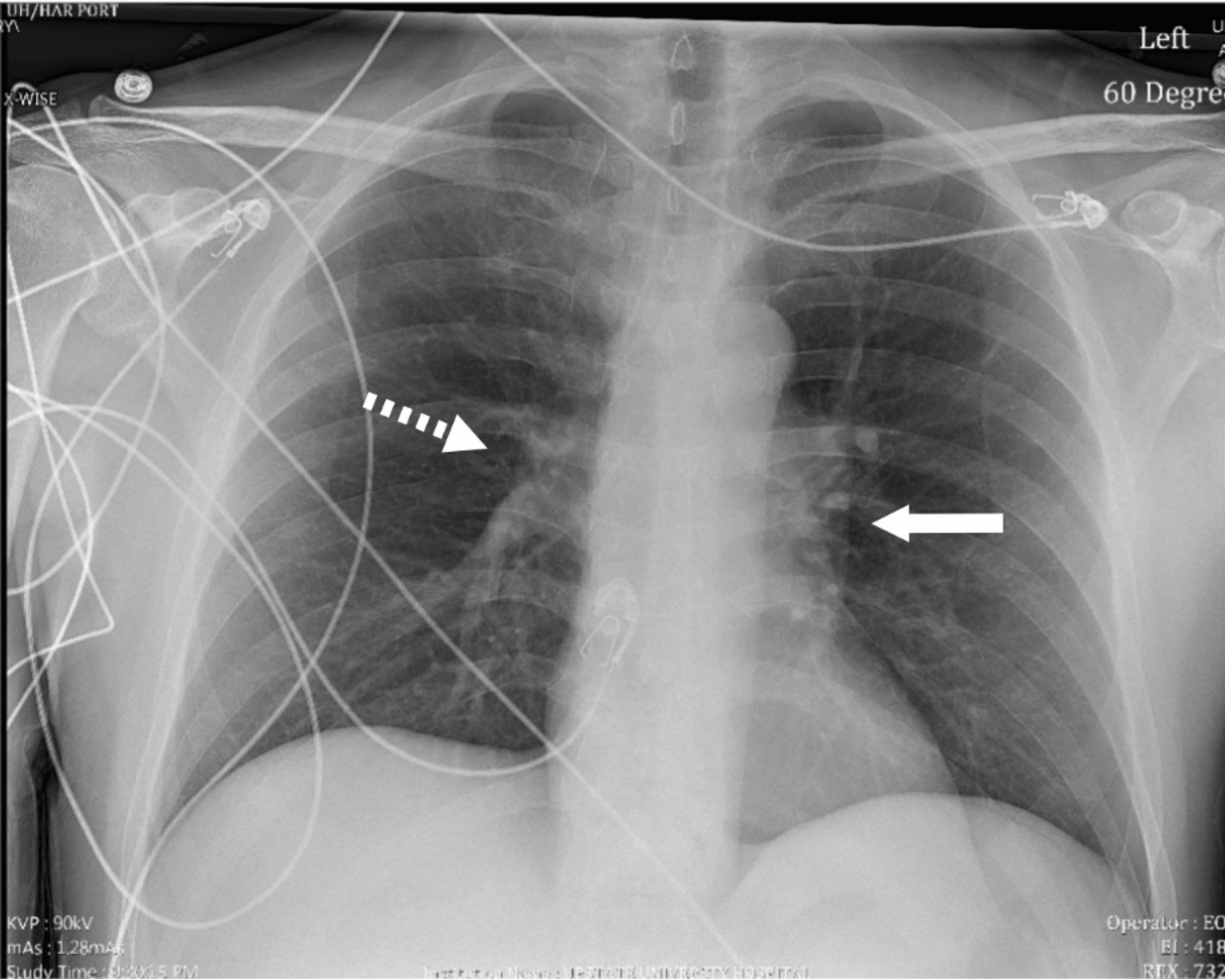
Radiograph of the chest showing left heart border prominence suggestive of enlarged pulmonary trunk (solid arrow) and pulmonary vascular congestion (dashed arrow).

Four days later, the patient presented to the ED again after a pre-syncopal episode at home. He was able to catch himself with no loss of consciousness or head trauma. He was found to be hemodynamically stable with negative orthostatic vital signs and unchanged ECG and labs from the prior visit. Chest X-ray (CXR) showed prominence of his left heart border, suggestive of an enlarged pulmonary trunk and pulmonary vascular congestion (Figure 2), a progression from an unremarkable CXR at his first ER encounter. Upon admission, his complex clinical picture included diplopia with nausea and vertigo, migratory visual deficit, finger pain and redness, upper extremity numbness and weakness, dyspnea, exercise intolerance, and presyncope.

**Figure 3:**
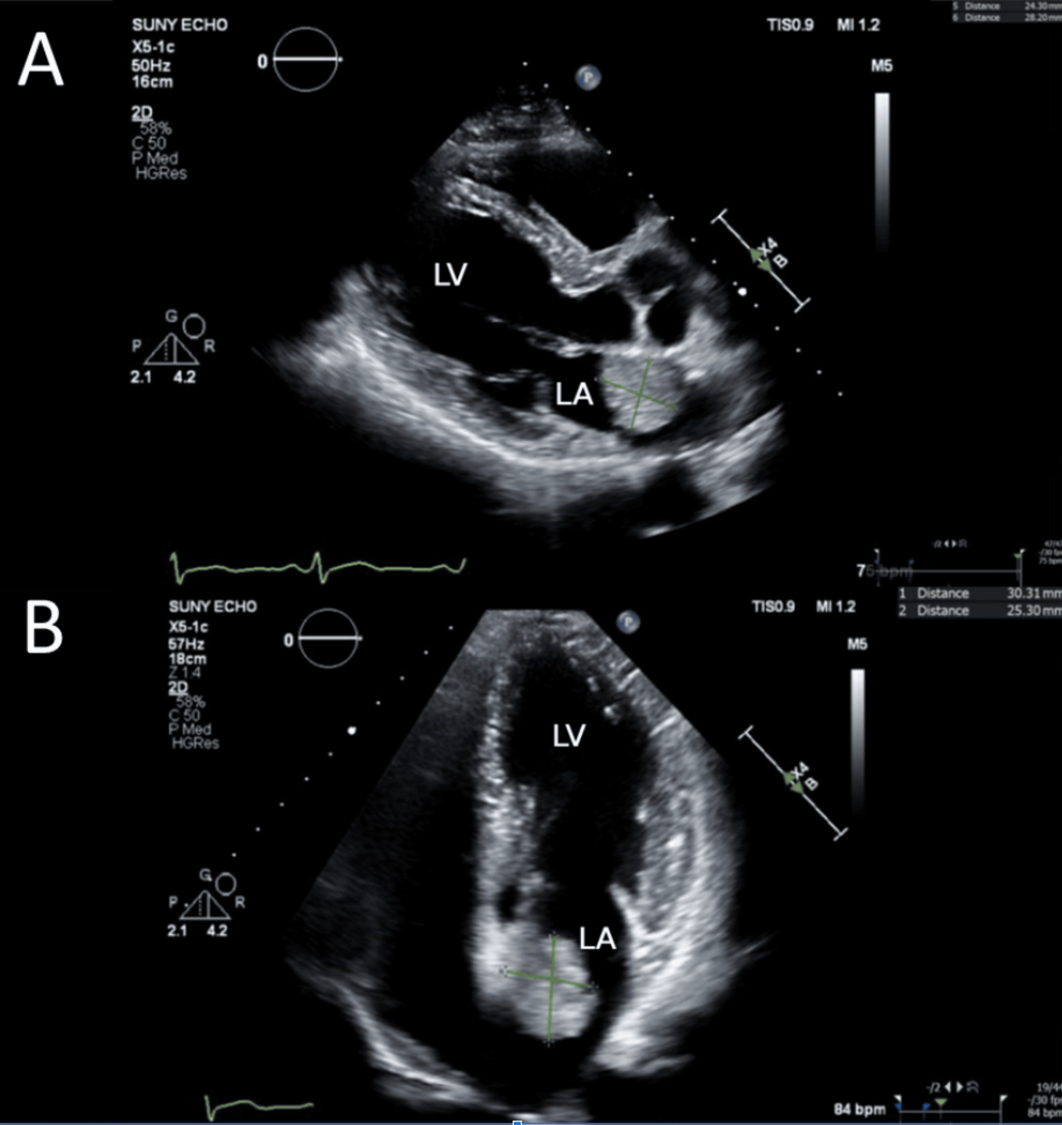
(A) Echocardiogram parasternal long axis view showing mass in the left atrium attached to the interatrial septum measuring 3.0 x 2.5 cm. (B) Echocardiogram apical four-chamber view showing redemonstration of the left atrial mass attached to the left atrial wall. LA: left atrium; LV: left ventricle.

Rheumatologic workup and infectious disease consultation were initiated on admission as the patient additionally had known contact with rare birds via his hobby of taxidermy, raising concern for zoonotic infection such as *Chlamydia psittaci*. However, the true culprit for his symptoms was revealed when an echocardiogram demonstrated a large mobile mass measuring 3 x 2.5 cm, possibly representing left atrial myxoma (Figure 3). No significant flow obstruction was noted.

**Figure 4:**
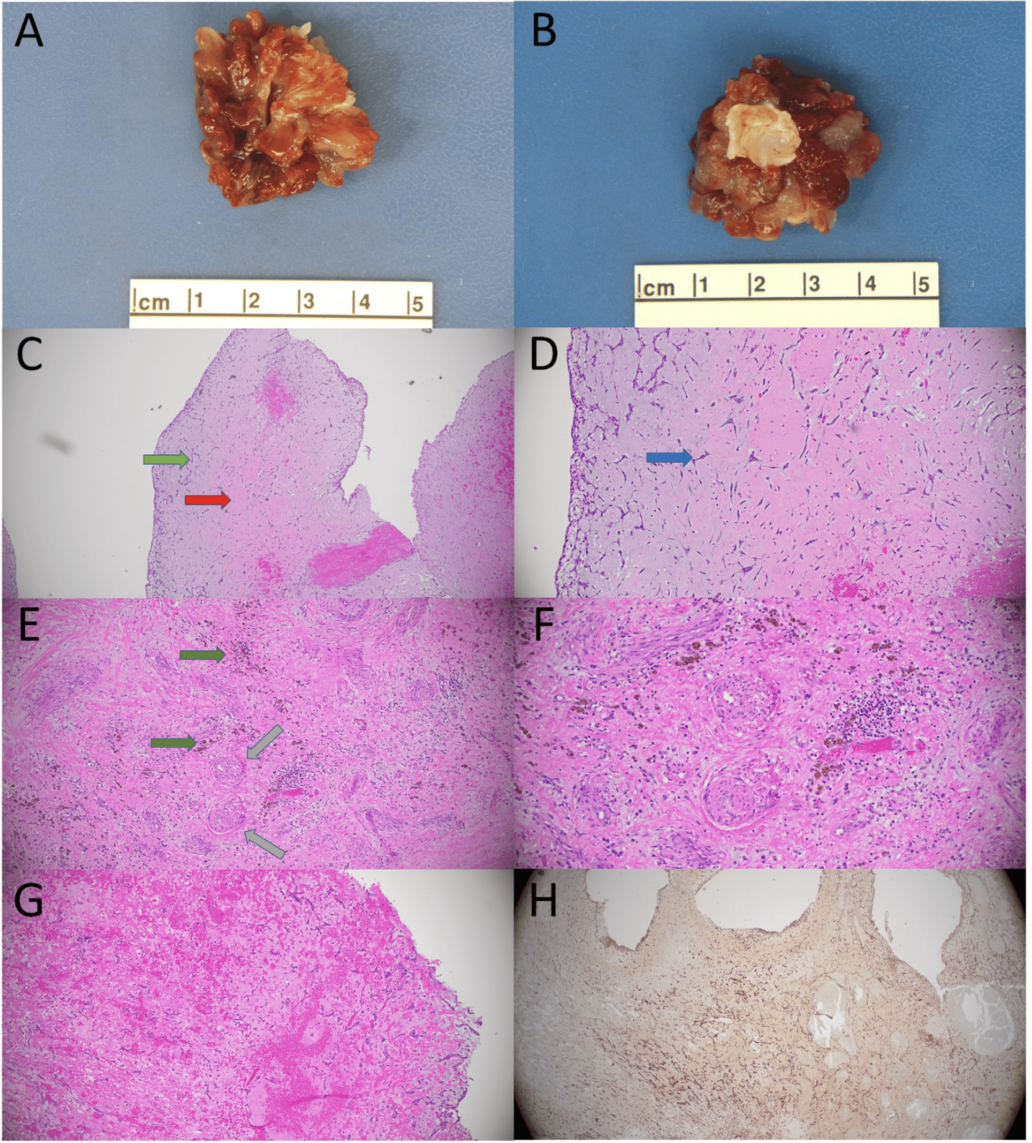
Gross anatomy and histology of cardiac myxoma. (A) Cardiac myxoma gross anatomy (3.8 x 3.4 x 2.4 cm), grossly papillary, mucoid, and with frequent variegations. (B) Cardiac myxoma’s tan-white fibromembranous stalk (1.4 x 1.1 cm). (C) Low power view showing prominent myxoid background (acellular gray-blue region, red arrow) with interspersed myxoma cells (green arrow). (D) Higher power view of picture C displaying characteristic bland and elongated myxoma cells. They have oval nuclei and eosinophilic cytoplasm (blue arrow). (E) Hemosiderin-laden macrophages demonstrating prior intratumoral hemorrhage (green arrows). Thickened arteries with medial hypertrophy are commonplace (gray arrows). (F) Higher power view of picture E, exhibiting prominent hemosiderin-laden macrophages and thickened vessels. (G) Diffuse hemorrhage with scattered myxoma cells throughout. (H) Calretinin immunohistochemistry stain is positive in the myxoma cells both cytoplasmically and nuclearly.

The patient underwent successful surgery with unremarkable postoperative visits. Resolution of diplopia, fevers, and dyspnea was noted at one-month postoperative follow-up. Pathology confirmed the diagnosis of a cardiac myxoma described as a 3.4 x 3.4 x 2.4 cm pink-red gelatinous mass with multiple projections and a 1.4 x 1.1 cm tan-white fibromembranous base (Figures 4A, 4B). Microsectioning showed a prominent myxoid background interspersed by characteristic myxoma cells with bland and elongated cellular architecture, oval nuclei, and eosinophilic cytoplasm (Figures 4C, 4D). Prominent hemosiderin-laden macrophages, demonstrating prior hemorrhage, were visible intratumorally. Thickened arteries with medial hypertrophy and prominent hemorrhage were observed diffusely (Figures 4E-4G). Calretinin stain was diffusely positive in myxoma cells, further confirming the diagnosis (Figure 4H).

## Discussion

Cardiac myxomas are relatively rare, with an incidence rate of 1.38 per 100,000 people per year [11]. They are the most common subtype of primary cardiac tumors, most frequently found in the left atrium [2,9]. Their initial clinical manifestations are remarkably nonspecific, including embolic phenomena (peripheral artery occlusion, embolic stroke), intracardiac obstruction (dyspnea, cough, angina, lower extremity edema, syncope), and constitutional disturbances (fatigue, fever) [3-8].

Our patient presented with an array of embolic (diplopia, vertigo), obstructive (dyspnea), and constitutional (fever) symptoms. The transient clinical nature and negative MRI findings suggest a clinical presentation most likely TIA, with small emboli lending to spontaneous recanalization. This proved clinically challenging as spontaneous self-responsive episodes of "vertigo" easily matched the clinical features of benign paroxysmal positional vertigo (BPPV). Regardless of other presenting symptoms, cryptogenic TIA/stroke syndromes require a complete imaging evaluation, including an echocardiogram. In his particular case, if TIA could not be ruled out, anticoagulation for his known atrial fibrillation should have been considered.

There were multiple factors in this case that obscured this underlying pathology. The patient’s primary complaints were neurologic, and myxoma most commonly presents with dyspnea and palpitations [2,5]. Interestingly, in this case, the patient had an MRI of the brain that did not reveal underlying pathology, even in the setting of suspected myxomatous embolization. Lack of an obvious infarct on initial MRI despite clinical symptoms consistent with embolization may suggest a requirement for higher sensitivity (3T MRI) or specialized techniques for image acquisition under appropriate clinical settings. This case also highlights the key role of echocardiography in the initial work-up of any suspected acute neurological symptoms. Additionally, this patient also had a prior diagnosis of paroxysmal atrial fibrillation, which may or may not have played a role in the suspected embolic phenomenon.

## Conclusions

The patient’s history of atrial fibrillation provokes further questions. The arrhythmia was diagnosed 15 years prior, raising the possibility of a potential connection with his eventual myxoma. Hypothetically, a slow-growing mass could disrupt typical cardiac conduction pathways or cause myofibrillar architectural disruption, causing atrial fibrillation and eventually manifesting as a fulminant atrial myxoma. Further study is warranted to investigate a connection between early-onset atrial fibrillation and the development of cardiac myxomas. Such a relationship would suggest one-time or routine echocardiography in the appropriate patient population.

This patient’s course highlights the challenges to early recognition and diagnosis of cardiac myxoma. He had initially presented to the ED with a subacute constellation of symptoms and reported frustration with his outpatient work-up. Some degree of anchoring bias was possible in his case, with a more common pathology like BPPV first considered. In our increasingly fast-paced healthcare, it is too conceivable to picture his first rushed clinic visit. Pathologies like atrial myxomas require time and thought to see the clinical forest for the trees. Early studies with TTE or chest CT would have been beneficial in understanding this patient. It was not until his presentation with presyncope that he was admitted, and the appropriate diagnostic tests were undertaken. At that time, structural heart disease was finally a serious consideration for being the etiology of his illness. This case supports the idea that clinicians should be sensitized to the varied and nonspecific multisystemic presentation of atrial myxomas (cardioembolic phenomena, obstructive symptoms, with nonspecific constitutional symptoms), which may facilitate early diagnosis and implementation of appropriate therapy.
